# Factors influencing decision regret regarding placement of a PEG among substitute decision-makers of older persons in Japan: a prospective study

**DOI:** 10.1186/s12877-017-0524-2

**Published:** 2017-06-28

**Authors:** Yumiko Kuraoka, Kazuhiro Nakayama

**Affiliations:** Graduate School of Nursing Science, St. Luke’s International University, 10-1 Akashi-cho, Chuo-ku, Tokyo, 104-0044 Japan

**Keywords:** Decision regret, Decision conflict, Decision-making, Feeding tube, Older person

## Abstract

**Background:**

A tube feeding decision aid designed at the Ottawa Health Research Institute was specifically created for substitute decision-makers who must decide whether to allow placement of a percutaneous endoscopic gastrostomy (PEG) tube in a cognitively impaired older person. We developed a Japanese version and found that the decision aid promoted the decision-making process of substitute decision-makers to decrease decisional conflict and increase knowledge. However, the factors that influence decision regret among substitute decision-makers were not measured after the decision was made. The objective of this study was to explore the factors that influence decision regret among substitute decision-makers 6 months after using a decision aid for PEG placement.

**Methods:**

In this prospective study, participants comprised substitute decision-makers for 45 inpatients aged 65 years and older who were being considered for placement of a PEG tube in hospitals, nursing homes and patients’ homes in Japan. The Decisional Conflict Scale (DCS) was used to evaluate decisional conflict among substitute decision-makers immediately after deciding whether to introduce tube feeding and the Decision Regret Scale (DRS) was used to evaluate decisional regret among substitute decision-makers 6 months after they made their decision. Normalized scores were evaluated and analysis of variance was used to compare groups.

**Results:**

The results of the multiple regression analysis suggest that PEG placement (*P* < .01) and decision conflict (*P* < .001) are explanatory factors of decision regret regarding placement of a PEG among substitute decision-makers.

**Conclusions:**

PEG placement and decision conflict immediately after deciding whether to allow PEG placement have an influence on decision regret among substitute decision-makers after 6 months.

**Electronic supplementary material:**

The online version of this article (doi:10.1186/s12877-017-0524-2) contains supplementary material, which is available to authorized users.

## Background

The American Geriatrics Society issued a position statement about feeding tubes in those with advanced dementia in 2014 that included the following statement: “Feeding tubes are not recommended for older adults with advanced dementia. Hand feeding is at least as good as tube feeding for the outcomes of death, aspiration pneumonia, functional status, and comfort. Tube feeding is associated with agitation, greater use of physical and chemical restraints, greater healthcare use due to tube-related complications, and development of new pressure ulcers” [1].

In Japan, the percentage of the population aged 65 years and over was 26.7% in 2015 [2], and the number of those with disturbance of swallowing is increasing. Many patients receive percutaneous endoscopic gastrostomy (PEG) and a tube feeding for artificial nutrition. A private-sector research institution in Japan [3] reported that PEG kit sales were above 100,000 units in 2005, and more than 700,000 people exchanged PEG kits in 2010. Surveys of the survival of older patients after PEG in Western countries found that 55% and 45% of patients survived more than 6 months and 1 year, respectively [4, 5]. On the other hand, in Japan, a survey of survival among 931 older patients after PEG found that 75% and 66% of patients survived more than 6 months and 1 year, and 50% and 25% of those who had feeding tubes survived 753 and 1647 days, respectively [6]. The survival rate of older patients after PEG in Japan is higher than in Western countries, and we think that is the reason why PEG placement is more common in Japan.

Few studies have examined decision-making for the use of feeding tubes [7–10]. Caregivers report little conversation about feeding tube decisions with medical staff (more than half of caregivers report no conversation or conversations of less than 15 min), and at times, families feel pressured to use feeding tubes [7]. Another study in Japan showed that many patients are not involved in the decision-making process because of cognitive problems, and most patients do not discuss their intentions regarding tube feeding with their family in advance; therefore, family members make all of the decisions with the support of physicians. In that study, physicians provided positive recommendations more frequently for the tube-feeding group (19/23, 83%) than for the withholding group (5/12, 42%) (*P* = .01) [8].

A previous study in Japan identified five factors that influence Japanese physicians’ decision to provide artificial hydration and nutrition (AHN) through PEG tubes: (1) the national health insurance system that allows elderly patients to become long-term hospital in-patients; (2) legal barriers with regard to limiting treatment, including the risk of prosecution; (3) emotional barriers, especially abhorrence of death by “starvation”; (4) cultural values that promote family-oriented end-of-life decision making; and (5) reimbursement-related factors involved in the choice of PEG [9].

A study of 4506 physicians who were members of the Japan Geriatrics Society found that only 6% responded that they did not have difficulty determining whether AHN should be introduced, and half responded that an ethical problem occurs when deciding to withhold AHN [10]. In Japan, since families and physicians often recognize cognitively impaired older patients to be unable to make their own decisions, physicians are generally relied upon for decisions involving AHN. A study on the perception of families of dementia patients who received placement of a PEG reported that 26.7% of 33 families wavered in their judgment of PEG between “good” and “not good”, and 13.3% felt it was “not good” [11]. Since older patients with cognitive impairment often cannot make choices for themselves, family members act as substitute decision-makers. The decision of family members often involves many conflicting facts and emotions and creates a heavy mental burden.

A tube feeding decision aid designed at the Ottawa Health Research Institute was specifically created for substitute decision-makers who must decide whether to allow placement of a PEG tube in a cognitively impaired older person who is unable to eat independently [12]. The decision aid contains information about the following topics: common causes of eating and swallowing problems in older persons with cognitive impairment; technical considerations regarding the placement of and how to use PEG tubes; the principles and process of substitute decision-making; the risks and benefits of tube feeding; the option of supportive care; and some considerations regarding future discontinuation of PEG tube feeding if the substitute decision-maker opts for an intervention. We developed and evaluated the Japanese version of this decision aid in a previous study with a before-and-after test design [13] (Additional file 1). In that study, substitute decision-makers showed significantly increased knowledge (*P* < .001) and decreased decisional conflict (*P* < .01) regarding long-term tube feeding after using the decision aid.

Decision aids for tube feeding in older patients have been shown to improve the quality of decision-making for substitute decision-makers. However, our previous study [13] focused on decision-making when they decided whether to introduce tube feeding and the factors that influence decision regret among substitute decision-makers were not measured after the fact. Therefore, the objective of this study was to explore the factors that influence decision regret among substitute decision-makers 6 months after using a decision aid for PEG placement.

## Methods

### Recruitment and data collection

Participants were recruited from all over Japan between January 2014 and August 2014. Participants were substitute decision-makers who were at the point of deciding whether to place a PEG tube in older family members and who obtained a decision aid that we developed. The decision aids were specifically prepared for substitute decision-makers charged with deciding whether to allow the placement of PEG tubes in cognitively impaired individuals aged ≥65 years and judged as unable to eat independently. We developed a website [14] that not only introduced the decision aid, but also enabled participants to request the decision aid. Decision aids could alternatively be obtained from medical staff.

**Fig. 1:**
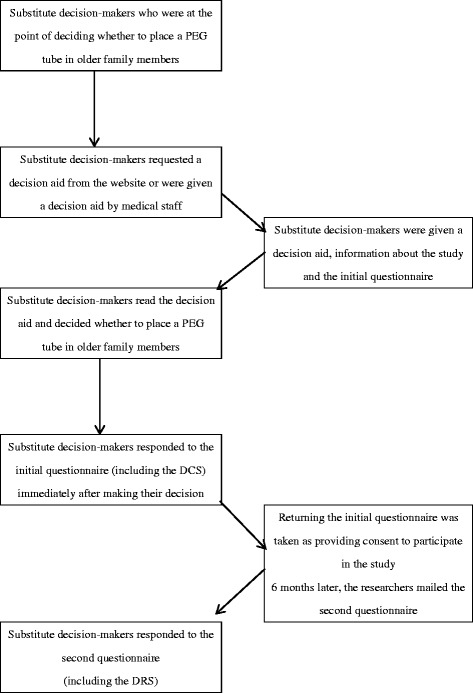
Recruitment and data collection

When substitute decision-makers requested a decision aid from the website, we mailed them the decision aid, information about the present study and the initial questionnaire. Substitute decision-makers who obtained the decision aid from medical staff were also given information about the study and a request to participate. We then mailed the initial questionnaire to those who consented to participate in this study. We asked substitute decision-makers to complete and return the initial questionnaire immediately after they decided whether or not to place a PEG tube. Returning the initial questionnaire was taken as providing consent to participate in the study. After 6 months, we mailed a second questionnaire to substitute decision-makers who returned the initial questionnaire. Those who returned completed the initial and second questionnaires were taken as study participants. (Fig. 1).

### Questionnaires

The questionnaires consisted of items on decisional conflict and decisional regret. Conflict with decision-making was assessed using the Decisional Conflict Scale (DCS) [15], which was translated into Japanese with the developer’s consent with reference to the Japanese version for women considering prenatal testing [16]. Based on the responses to 16 questions, this scale evaluates five domains of decisional conflict: (1) certainty regarding choices; (2) feeling informed; (3) feeling clear about values; (4) feeling supported in decision-making; and (5) quality of the decision. Each question was rated on a 5-point Likert scale, with higher values representing greater decisional conflict. The DCS can be evaluated both before and after decision-making and was included only in the initial questionnaire in this study. The validity and reliability of the Japanese version of the DCS have been confirmed [17].

Decisional regret was measured using the Decisional Regret Scale (DRS) [18], which measures distress or remorse after a health care decision and reflection on a specific past decision. The DRS includes a five-item scale and responses to each item were given on a 5-point Likert scale ranging from 1 (“I strongly agree”) to 5 (“I strongly disagree”), with higher values representing greater decisional regret. The validity and reliability of the Japanese version of the DRS have been confirmed [19].

### Study design

A prospective study was conducted to evaluate the DCS and DRS. Substitute decision-makers completed the initial questionnaire, which included the DCS, immediately after making their decision. The substitute decision-makers were contacted again 6 months after the initial contact and asked to respond to a second questionnaire that included the DRS.

### Statistical analysis

Normalized scores were evaluated and multiple regression analysis was used to identify explanatory factors that influence decision regret among substitute decision-makers. We set patient characteristics (gender, survival, PEG placement or not, patient can convey own wishes or not), substitute decision-maker characteristics (age, relationship to the patient), physician’s recommendation for the patient to receive PEG placement or to withhold PEG, and substitute decision-makers’ decision conflict as independent variables. We set the DRS score as the dependent variable. The independent variables and dependent variable were set with reference to previous studies [7, 8, 10, 13].

## Results

### Enrollment

One hundred fifty two substitute decision-makers obtained the decision aid from the website and one substitute decision-maker obtained the decision aid from medical staff in charge of the patient. All decision-makers were approached by the study team. Of these, a total of 45 (29.4%) substitute decision-makers agreed to participate in the study. Forty-five (100%) of 45 substitute decision-makers responded to the follow-up questionnaire.

### Patient and substitute decision-maker characteristics

Table 1 shows the characteristics of the patients enrolled in the study. The mean age ± standard deviation (SD) of the patients was 84.1 ± 8.3 years; 24 were female. The diagnostic indications for PEG placement included aspiration pneumonia (*n* = 17), dementia (*n* = 11), cerebral infarction (*n* = 11), Parkinson disease (*n* = 2), depression (*n* = 2), pneumonia (*n* = 2), intractable neurological diseases (*n* = 2), cancer (*n* = 1) and other disease (*n* = 13). The settings of the patients were an acute care hospital (*n* = 16), mixed care hospital (*n* = 10), long-term care hospital (*n* = 8), nursing home (*n* = 8), and patient’s home (*n* = 3). The level of physical activity of the patients was bed-bound but cannot toss and turn (*n* = 24), bed-bound but can toss and turn (*n* = 10), use a wheelchair mainly (*n* = 4), ambulant (*n* = 4), and other level (*n* = 3). The communicative ability of the patients was cannot communicate sometimes (*n* = 22), cannot communicate at all (*n* = 16), and can easily communicate (*n* = 7).

**Table 1:** Characteristics of patients and substitute decision-makers (*n* = 45)

Patients			
Mean age (years)		84.1 ± 8.3	
Female		24	53.3%
Diagnosis	Aspiration pneumonia	17	
	Dementia	11	
	Cerebral infarction	11	
	Parkinson disease	2	
	Depression	2	
	Pneumonia	2	
	Intractable neurological diseases	2	
	Cancer	1	
	Other disease	13	
	(multiple answers allowed)		
Setting	Acute care hospital	16	35.6%
	Long-term care hospital	8	17.8%
	Mixed care hospital	10	22.2%
	Nursing home	8	17.7%
	Patients’ home	3	6.7%
Physical activity	Bed-bound (cannot toss and turn)	24	53.3%
	Bed-bound (can toss and turn)	10	22.2%
	Primarily use a wheelchair	4	8.9%
	Ambulant	4	8.9%
	Other level	3	6.7%
Communicative ability	Can easily communicate	7	15.6%
	Cannot sometimes communicate	22	48.9%
	Cannot communicate at all	16	35.6%
Ability to convey wishes about PEG placement	Able to convey own wishes	12	26.7%
	Unable to convey own wishes	28	62.2%
	Neither	5	11.1%
Wishes regarding PEG placement	Place PEG	7/12	58.3%
	Withhold PEG	5/12	41.7%
Substitute decision-makers			
Mean age (years)		62.1 ± 10.5	
Male		25	55.6%
Relationship to the patient	Child	24	53.3%
	Spouse	11	24.4%
	Son/daughter-in-law	5	11.1%
	Nephew/niece	1	2.2%
	Brother/sister-in-law	1	2.2%
	Other relative	3	6.7%
How decision aid was obtained	Website	44	97.8%
	Medical staff	1	2.2%
Read the contents of the decision aid	All	43	95.6%
	Some	1	2.2%
	None	1	2.2%
Consulted with	Physician in charge of the patient	11	
	Care manager	6	
	Nurse	5	
	Physician not in charge of the patient	2	
	Social worker	1	
	Nobody (multiple answers allowed)	23	
Decision in accordance with the patient’s wishes	PEG placed	7/7	100%
	PEG withheld	5/5	100%

Twelve of 45 patients were able to convey their wishes. Seven of these 12 patients wished to receive PEG and five wished to not receive PEG. Of these, seven of seven substitute decision-makers decided for PEG placement and five of five substitute decision-makers decided to withhold PEG, respectively; therefore, the decisions of all 12 of these substitute decision-makers were in accordance with the wishes of the patient.

The mean age ± SD of the substitute decision-makers was 62.1 ± 10.5 years; 25 were male. Substitute decision-makers had the following relationships to the patients: child (*n* = 24), spouse (*n* = 11), son/daughter-in-law (*n* = 5), nephew/niece (*n* = 1), brother/sister-in-law (*n* = 1), and other relative (*n* = 3). Forty-four of 45 substitute decision-makers found the website that we developed and requested that we mail a decision aid to them. One of 45 substitute decision-makers was handed a decision aid by a medical staff member. Forty-three of 45 substitute decision-makers read all the contents of the decision aid. One of 45 substitute decision-makers read some of the contents of the decision aid. One of 45 substitute decision-makers did not read the decision aid. Eleven of 45 substitute decision-makers consulted with the physician in charge of the patient regarding the decision aid. Six of 45 substitute decision-makers consulted with a care manager. Five of 45 substitute decision-makers consulted with a nurse. Two of 45 substitute decision-makers consulted with a physician not in charge of the patient. One of 45 substitute decision-makers consulted with a social worker. Twenty-three of 45 substitute decision-makers consulted with nobody.

### Physician’s recommendation

We asked substitute decision-makers whether the physician recommended for the patient to receive PEG placement, to not receive PEG placement, or made no recommendation. Twenty-five substitute decision-makers answered that the physician recommended PEG placement, five substitute decision-makers answered that the physician did not recommend PEG placement, and 15 substitute decision-makers answered that the physician did not make any recommendation. Physicians provided positive recommendations more frequently for the PEG placement group (18/20, 90%) than for the withholding group (7/25, 28%) (*P* < .001).

### Decisions and functional status

**Fig. 2:**
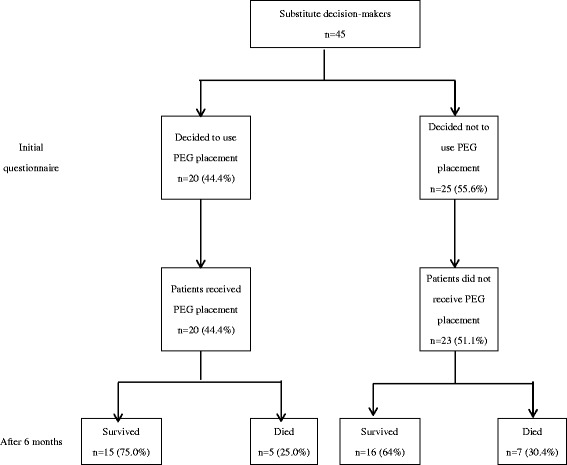
Decisions and functional status of patients

Twenty (44.4%) of 45 substitute decision-makers had decided to use PEG placement when they completed the initial questionnaire, and 20 (44.4%) of these patients received PEG placement. Twenty-five (55.6%) of 45 substitute decision-makers had decided not to use PEG placement when they completed the initial questionnaire, and 23 (51.1%) of these patients did not receive PEG placement. During the follow-up period, 15 (75%) of the 20 patients who received PEG placement survived, and 16 (69.6%) of the 23 patients who did not receive PEG placement survived (Fig. 2).

### Multiple regression analysis

The results of the multiple regression analysis suggest that PEG placement (*P* < .01) and decision conflict (*P* < .001) of substitute decision-makers are explanatory factors of decision regret among substitute decision-makers (Table 2).

**Table 2:** Results of the regression analysis: final models

		Bivariate regression analysis			Multiple regression analysis		
Variable	Mean	β	F	P	β	F	P
Dependent variable: Decision regret of substitute decision-maker							
Patients							
Gender/male	33.86	−0.209	1.866	0.179	0.171	0.521	0.476
Gender/female	30.42						
Survival/survived	30.83	0.034	0.048	0.827	−0.207	0.547	0.465
Survival/died	34.05						
PEG placed	38.19	−0.423	8.732	0.005	0.796	9.925	0.004
PEG withheld	24.82						
Patient can convey own wishes	25.00	−0.427	1.514	0.226	−0.411	1.873	0.182
Patient cannot convey own wishes	33.13						
Substitute decision-makers							
Gender/male	30.44	0.209	1.866	0.179	−0.071	0.068	0.797
Gender/female	33.61						
Age		0.010	0.004	0.948	0.015	1.719	0.200
Relationship/child of patient	30.40	−0.099	0.409	0.526	0.126	0.237	0.630
Relationship/not child of patient	34.16						
Consulted with							
Medical staff	35.81	0.132	0.695	0.409	−0.166	0.215	0.646
Nobody	28.38						
Physician’s recommendation							
Either withhold or place PEG	35.20	−0.252	2.787	0.103	0.194	0.424	0.520
No recommendation	24.10						
Decision conflict of substitute decision-makers		0.583	21.091	<0.001	0.625	21.945	<0.001

Gender: 1 = male, 2 = female

Survival: 1 = survived, 2 = died

PEG: 1 = PEG placed, 2 = withheld

Patient’s wishes: 1 = patient can convey own wishes, 2 = patient cannot convey own wishes

Relationship: 1 = child of patient, 2 = not child of patient

Consulted with: 1 = medical staff, 2 = nobody

Physician’s recommendation: 1 = either withhold or place PEG, 2 = no recommendation

## Discussion

In this study, only 24.4% of the patients suffered from dementia and 64.5% of the patients could easily communicate or could communicate most of the time. The level of dementia of the patients who participated in this study varied. Of the patients who received PEG placement, 75% survived more than 6 months. Our finding for mortality was similar to the results of a previous survey of survival of older patients after PEG placement in Japan [6]. Some patients in that study had dementia (15% had severe dementia, 3% had mild dementia, 14% had other types of dementia), but the prognosis did not differ between this study and that study [6].

The results of the present study showed physicians provided positive recommendations more frequently for the PEG placement group than for the withholding group (*P* < .001). We believe it is possible that substitute decision-makers might have decided to use PEG placement because they felt pressure from the physician. The American Geriatrics Society stated that, “Institutions such as hospitals, nursing homes, and other care settings should promote choice, endorse shared and informed decision-making, and honor individuals’ preferences regarding tube feeding. They should not impose obligations or exert pressure on individuals, or providers to institute tube feeding” [1]. We think it is important to educate medical providers about appropriate end-of-life care for old people for example, the introduction of feeding tubes is not typically performed to prolong life, prevent aspiration pneumonia, heal pressure ulcers, or improve quality of life [20–22].

The results of the present study showed that decision regret among substitute decision-makers of patients who received PEG placement was significantly higher than that of those who did not receive PEG placement. The reason why many substitute decision-makers choose PEG placement is because it is difficult to accept the patient’s death by starvation [23, 24]. A previous study in Japan [23] showed substitute decision-makers worried about whether PEG placement was good for the patients; the results of the present study ware similar. It is important that medical staff support substitute decision-makers carefully if they decide to use PEG placement, and substitute decision-makers should receive ongoing education about the natural senile process and the natural progression of dementia, which includes eating difficulties.

Substitute decision-makers who had higher decision conflict when they chose or withheld PEG placement also had higher decision regret after 6 months. Endo et al. [25] proposed that medical staff and family should confirm the patient’s intentions in the early stages of dementia. We believe the decision conflict of substitute decision-makers will decrease if they can make decisions based on the patient’s intentions. In this study, 12 of 45 (26.7%) patients were able to convey their own wishes and their substitute decision-makers could act in accordance with their wishes. We believe if patients can convey their own wishes, the patient and family can share decision-making. On the other hand, although 29 of 45 (64.5%) patients could easily communicate or could communicate most of them, substitute decision-makers indicated that only 12 of 45 (26.7%) patients could convey their own wishes. It is possible that family and medical staff might not make enough of an effort to determine the patient’s wishes. Family and medical staff should support patients in making their own decisions and make an effort to hear the patient’s wishes when they are deciding whether to use or withhold PEG placement.

We found that 97.8% of substitute decision-makers obtained the decision aid by themselves through the website and 95.6% read all the contents of the decision aid. We think that most substitute decision-makers in the present study had high health literacy. Although a previous study showed that a decision aid for feeding options in advanced dementia improved the frequency of substitute decision-makers’ communication with medical providers [26], 51.1% of substitute decision-makers in the present study did not consult with medical staff regarding the decision aid. This seems partly because the Japanese cultural environment supports physicians’ paternalism and substitute decision-makers hesitate to approach physicians. It is important that substitute decision-makers communicate with medical providers regarding the decision aid to discuss the probable wishes of the patient and the best choice for that specific patient, especially when the patient’s wishes are unknown.

The present research has some of important limitations. First, because the number of participants was small, generalization of the results is limited. The number of participants should be increased to analyze the explanatory factors of decision regret among substitute decision-makers in more detail.

Second, the elderly individuals investigated in this study had varying levels of cognitive function, as there were cases in which patients were able and unable to convey their wishes regarding PEG placement. Therefore, it can be inferred that there were various cases, from those in which families made decisions by respecting the wishes that were clearly indicated by the patients, to those in which the wishes of the patients were unclear and families made decisions by surmising the patients’ wishes.

Third, the subjects of this study were limited to substitute decision-makers who used our decision aid for decision-making; we did not compare decision regret among substitute decision-makers who made decisions without using the decision aid. It is necessary to compare decision regret of both types of substitute decision-makers to determine the efficacy of this decision aid over the long term.

Fourth, the most frequent settings of patients in the present study were hospitals and nursing homes. The setting of medical treatment in Japan is increasingly shifting from hospitals to the home. Thus, it is also necessary to further evaluate decision regret for patients receiving care at home.

## Conclusions

The present study showed that PEG placement (*P* < .01) and decision conflict (*P* < .001) of substitute decision-makers immediately after deciding whether to use PEG placement in a cognitively impaired older person have an influence on decision regret after 6 months. Further research comparing substitute decision-makers who made decisions about placement of a PEG both with and without the decision aid is needed to compare decision regret among substitute decision-makers and to analyze the long-term efficacy of this decision aid.

## Additional file

Additional file 1 English version of decisional aid. (PDF 9372 kb)

## Abbreviations

AHN: artificial hydration and nutrition
DCS: Decisional conflict scale
DRS: Decision regret scale
PEG: percutaneous endoscopic gastrostomy
