# Supplementary material for: Factors influencing decision regret regarding placement of a PEG among substitute decision-makers of older persons in Japan: a prospective study
Source: BMC Geriatr. 2017 Jun 28;17:134. doi: 10.1186/s12877-017-0524-2 (PMC5490187; doi:10.1186/s12877-017-0524-2)
Supplement: Additional file 1 — English version of decisional aid. (PDF 9372 kb) [file 12877_2017_524_MOESM1_ESM.pdf]

A booklet for substitute decision makers
Making Choice: Long term feeding tube placement in elderly patients

ご本人に代わって意思決定を行う方のための小冊子

高齢者が栄養チューブをつけて長期的に使うこと

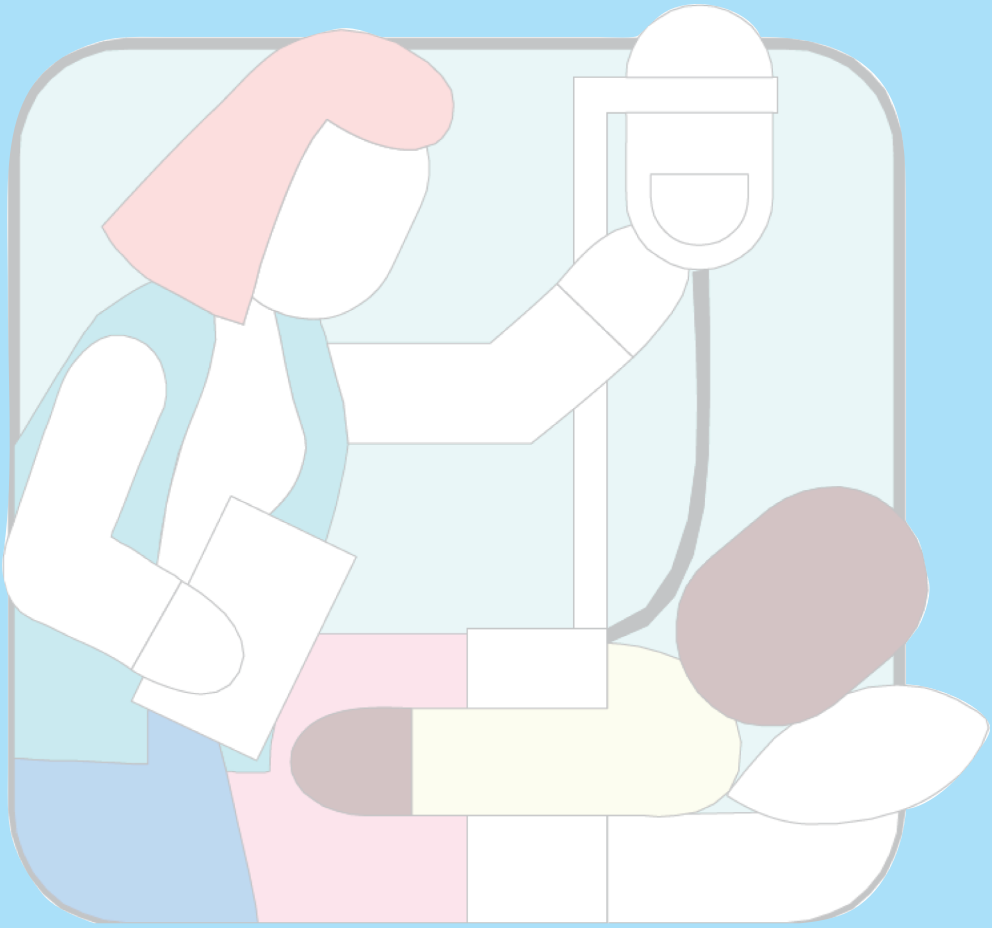

はじめに

この小冊子は高齢者に栄養チューブをつけて長期的に使うことについて、あなたが判断することを助けるものです。冊子の内容を検討していくうちに、あなたの大切な家族・近親者または友人に栄養チューブをつけることの利点と欠点、代理意思決定について学べるようになっています。

この冊子の記述内容を裏付ける研究結果には、○○[1] のように番号が振られています。冊子の後半部 17 ページ以降に参考文献の一覧表があります。

対象者

・現在、**自分自身の健康管理に関する判断を行う事が出来ない高齢者に代わって意思決定を行う**
・上記高齢者に胃ろう（PEG）もしくは空腸ろう（j-tube）として知られている栄養チューブをつけて長期的に使うことが必要かどうか判断しなければならない人

この小冊子から分かること

- 代理意思決定
- 摂食・嚥下障害
- 治療の選択肢
- 栄養チューブ
- 栄養チューブをつけることの利点と欠点
- 判断の方法

Welcome
This workbook have been designed to prepare you for a decision about placing a feeding tube in an elderly patient. As you go trough the booklet, you will learn about substitute decision making as well as the advantages and disadvantages of placing a feeding tube in your friend or family member.
Research studies that support statements in this booklet are referenced by numbers like this:[1].
The complete list of references is at the back of the booklet, starting on page17.

The workbook is for you if:
ou are the substitute decision maker for an older person who is currently unable to make his/her own hearth care decisions.
you need to decide whether the person should have a long term feeding tube known as a gastrostomy tube (PEG) or a jejunostomy tube (j-tube).

You will learn about:
eating and swallowing problems, feeding tubes, substitute decision-making, advantages and disadvantages of feeding tube placement, treatment options, how to decide

「代理意思決定[1]」とは何でしょうか？

- 健康管理に関する判断を自分自身で行う事が出来ない人のために意思決定を行うことです。
- 本人が望むと思われることが、同じ立場に立った場合にあなた自身が選択することとは異なる場合があります。本人の望むと思われることを、最も優先して考えることが、本人の尊厳を守ることにつながります。
- 代理意思決定は非常に難しく精神的な負担となることがあります。

「意思決定を行う代理人」には誰がなるのでしょうか？

- 以前本人から指名されていた人（**健康管理に関する委任権**を持つ人）
- 最近親者
- 任命された後見人

どのような段階を経て
代理意思決定が行われるのでしょうか？

1） これまでに本人が示した意思を考慮しましょう。本人の意思を確認できるものには以下のものがあります。

- リビングウィルまたは「事前指示書」
- これまでに本人が代理人や他の人たちと話し合ったこと

これらの内容は、たとえあなたが同意出来なくとも、本人の意思として尊重されるべきものです。

2） 本人が健康だった時に本人が持っていた価値観についてすべてわかる範囲で検討します。あなたが知る範囲で、本人が今の状況に置かれた時に栄養チューブをつけることを選択すると思いますか？それともしないと思いますか？これを「代理判断」と呼びます。

3） これまでに本人の意思表示がなく、本人が望むと思われることが判断出来ない場合、本人にとっての最善の利益を、以下のことを考慮したうえで検討します。

- 栄養チューブによる利点と考えられる事
- 栄養チューブによる欠点と考えられる事
- この判断が本人の生活の質 (「生活の質」については 7 ページを参照］に及ぼす影響

意思決定を行う代理人の
書面による同意なしで栄養チューブが
つけられることはあるのでしょうか？

ありません。

What is "substitute decision making"?

- decision for others who are unable to make their own hearth care decisions
- what the patient would want may not be the same as what you would choose for yourself in the same situation
- substitute decision making can be very difficult and emotional

Who becomes a "substitute decision maker"?

- a person previously named by the patient (someone who has power of attorney for hearth care)
- next-of-kin
- appointed guardian

What are the steps involved in substitute decision making?

1) Consider the previously expressed wishes of the patient from either:
   - living will (sometimes called an "advance directive")
   - previous discussions the patient had with you and/or others

These wishes should be respected, even if you do not agree with them.

2) Consider all you know about the values of your patient when she was well. From what you know do you think she would choose to get a feeding tube in this situation or not? This is called "substituted judgement".
3) If these are no previously expresses wishes and you cannot judge what your patient would want, consider what is in his "best interests".
   - what are the possible advantages of tube feeding
   - what are the possible disadvantages of tube feeding
   - how will this decision affect his quality of life

Can a feeding tube be placed without the written consent of the substitute decision-maker?

- No

摂食（食べること）・嚥下（飲み込むこと）の障害はなぜ起こるのでしょうか？

- **嚥下を適切に行うために必要な筋肉や神経の損傷：**
  - 脳卒中
  - パーキンソン病
  - 筋萎縮性側索硬化症
- **自分で食事が出来なくなる病気：**
  - アルツハイマー病
  - その他の認知症
- **食道（口と胃をつなぐ管）がふさがる病気：**
  - 食道がん
  - 食道狭窄
- **重度の食欲不振や食事に対する興味が無くなる病気：**
  - 大うつ病

摂食・嚥下障害は高齢患者にどのような影響を及ぼすのでしょうか？

身体的影響

誤嚥：本人の眠気が非常に強い場合や飲み込むことに必要な神経や筋肉に障害がある場合、食物や唾液などが気管に入ってしまうがあります。これが肺炎の原因となる可能性があります。

栄養状態の不良：本人に起こること

- 体力がなくなります
- 体重が減少します
- 周りで起こっていることに対する認識力が低下します
- 急病からの回復が遅れます

空腹感やのどの渇き：意識がはっきりしている場合は、空腹感やのどの渇きを感じることがありますが、意識がはっきりしていない場合では、空腹感やのどの渇きを感じる可能性は低くなります。[2]

摂食・嚥下障害は高齢患者の家族にどのような影響を及ぼすのでしょうか？

精神的影響

家族・近親者や友人の方は、ご本人の重い病気を受け止める事を難しいと感じるかもしれません。近しい人が十分な食事をとれない状態を見るのがつらいと感じることや、ご本人が空腹感やのどの渇きを感じているのではないかと心配になることがあります。

社会的影響

- 食事は社会的なものであり、介護をする人にとっても重要なものです。
- ご本人の食事を介助することは、ご本人とコミュニケーションをとる 1 つの方法であり、介護する人にとって楽しみでもあります。
- ご本人が自分の手で食事をとることが出来ず、介助することもできない場合、家族は個人的な意思の疎通が出来なくなったと感じることがあります。しかし、ご本人と交流する方法は他にもあります。

Why do people develop eating and swallowing problems?
Damage to the muscles and nerves needed for proper swallowing,
- Possible causes are:
- Stroke
- Parkinson' s disease
- Amyotrophic lateral sclerosis

Inability to eat independently because of:
- Alzheimer's disease
- other dementias

Blockage of the esophagus (the tube that goes from the mouth to the stomach)
- cancer of the esophagus
- stricture

Severe loss of appetite or interest in eating:
- major depression

How do eating and swallowing problems affect older patients and those close to them?

PHYSICAL

Aspiration: Food or saliva may be inhaled into the lungs if the patient is very drowsy or if he has problems with the nerves or muscles needed to swallow. This may result in lung infections.

Poor nutrition: The patient will:
- become weaker
- lose weight
- become less aware of what is going on
- not recover as quickly from a sudden illness

Comfort: A patient who is very aware may feel hungry and thirty. Patients who are not very aware may not feel hunger or thirst.

EMOTIONAL

Friends and family may find it difficult to accept a patient's serious illness. They may find it hard to see a person close to them not eat enough. They might feel worried that the patient may feel hunger or thirst.

SOCIAL
- Eating is social and symbolic of care giving.
- Helping a patient to eat can be a pleasant way to interact with him or her.
- If a patient cannot be hand fed, the family may feel a loss of this personal interaction. However, other ways of socializing with him or her are always possible.

経皮的内視鏡下胃瘻造設術（PEG）による胃ろうとは何でしょうか？

- 摂食 (食べること) 障害のある人の胃に直接つけるチューブのことです。
- これは**治療の選択肢 (医療行為) の一つです。**
- **経皮的** - 皮膚を通します。
- **内視鏡的**-医師はカメラが付いたチューブ（内視鏡）を本人の胃に挿入し、胃ろうチューブを正しい位置につけることができるようにします。
- **胃瘻造設術** - 腹部に開けた小さな穴を通して胃にチューブをつける手術のことです。

＊　胃ろうとは別の空腸ろうと呼ばれる栄養チューブをつけることが提案されることがあります。空腸ろうと胃ろうとは若干異なります。空腸ろうについては、医師に確認して下さい。

チューブはどのように設置されるのでしょうか？

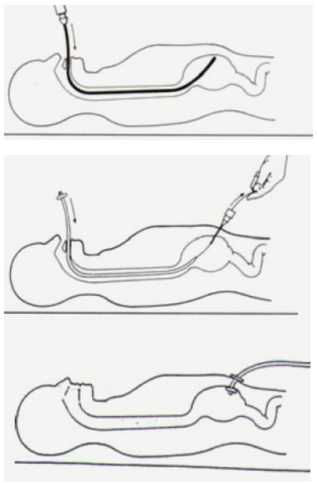

- 本人に軽い鎮静剤を投与します。
- 口から内視鏡を胃に入れます。少し不快感があるかもしれませんが、痛みはありません。内視鏡を入れることは、チューブをつけるのに最適な場所を確認するために必要なことです。
- 局所麻酔剤で患者の腹部の皮膚を麻痺させ、小さな切開部を作ります。胃ろうチューブを口から挿入し、腹部の切開部より体外に引き出します。
- この処置には約 30 分かかります。
- 食道が何らかの原因により塞がれ、内視鏡が挿入できない場合があります。その際は外科手術により栄養チューブを設置します。

栄養チューブでどのように食べ物を取るのでしょうか？

- 流動食をバッグに入れ、チューブを通して胃に送ります。
- 流動食は本人がバランスの取れた食事をとることができるようにつくられた液体です。ミルクセーキのようなものです。
- ほとんどの場合、通常の食事の時間にチューブを通して食事をとることになります。これには約 1 時間かかります。同量の流動食を 24 時間かけてゆっくりと持続して取る場合もあります。
- 水分や内服薬もチューブを通して取ることになります。

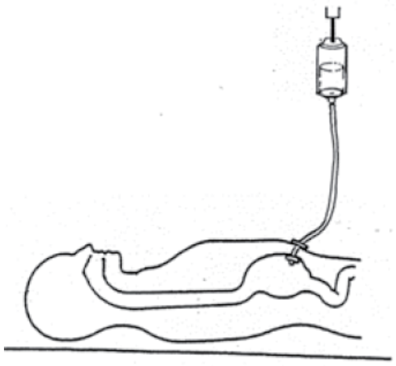

What is a percutaneous endoscopic gastrostomy (PEG) tube?

A tube placed directly into the stomach of someone with eating problems

An optional medical treatment

Percutaneous -through the skin

Endoscopic- a doctor will put a tube with a camera in it (an endoscope) down into the patient's stomach to help guide the tube into the correct spot

Gastrostomy- a procedure where a tube is put into the stomach through a small hole in the abdomen

How is the tube put into place?

The patients is mildly sedated (not put to sleep).

The endoscope is placed through the mouth and into the stomach. This can be a bit uncomfortable, but it dose not hurt. It is needed to see where the best place is to put the tube.

The patient is given a local anaesthetic to freeze the skin on the abdomen so that a small cut can be made. The tube is inserted through the mouth and pulled out through the opening in the abdomen.

The procedure takes about 30 minutes.

Sometimes it is not possible to insert the endoscope because the esophagus is blocked by a growth or tumour. In the these cases, the feeding tube would be placed surgically.

How dose the person with the feeding tube get their food?

Liquid food is put into a bag and then delivered into the stomach through a tube.

The food is a commercially prepared liquid that provides a balanced diet for the patient. It is something like a milkshake.

Most patients will be fed through the tube as usual meal times. The feeding will take about one hour. Some patients will receive continuous feeding in which the same amount of food is given, but at a slower rate over 24hours.

Medications as well as water will also be given through the tube.

チューブの管理に必要な事は何でしょうか？

- チューブは抜けないように固定されていますが、ご本人が無意識のうちにチューブを引き抜かないように注意する必要があります。
- 看護師 (自宅で療養する場合は家族) がチューブの漏れや詰まりを調べ、流動食が適切にとられているかを確認します。
- 看護師 (自宅で療養する場合は家族) が一日に一回以上チューブの周りを清潔にし、周囲の皮膚を調べます。
- 流動食の量は、本人が消化・吸収できる範囲で医療者が調整します。
- 通常、6 ヶ月～ 1 年でチューブを取りかえる必要があります。

チューブをつけた人は 口から全く食べられないのでしょうか？

いいえ、本人の飲み込む力の状況によっては、飲み込みやすいもの (ゼリーなど) を少量、口から食べることができます。本人が口から食べられるのか否かは医療チームに相談しましょう。

チューブをつけた人は 寝たきりになってしまうのでしょうか？

いいえ、チューブを使用していない時、体の外に出ている部分は外すことが出来るので本人の通常の行動を妨げることはありません。

チューブを用いた栄養法を 導入するうえで心配なこと

チューブを用いた栄養法を導入するうえで、考えられることには大きく分けて 2 種類あります。

- 栄養チューブ自体によって特定の合併症が生じる可能性
- 栄養チューブについての話し合いが行われる際に最もよく話題にのぼること

◆ **生存の可能性**

◆ **誤嚥**（食物などが気管に入ってしまうこと）

次の数ページでは、これらの結果について考え、チューブを用いた栄養法についての利点、欠点、その他の考慮すべき事柄について説明しています。

What is involved in the care of the tube?

- Care must be taken not to pull out the tube.
- The nurse will check for tube leakage, blockage and will make sure that the food is going in properly.
- The nurse will clean around the tube at least once a day and check the surrounding skin.
- The tube will usually need to be replaced within six months to one year.

Will the person with a gastrostomy tube have to stay in bed?

- No, the tube is very portable. When the tube is not in use, it will not restrict the patient's usual activities.

Possible health outcome from feeding tubes

Tube feeding is a medical treatment that can have a variety of possible health outcomes or consequences. These outcomes can divided into two types:

- Specific complications from the feeding tube itself
- General health outcomes that most commonly come up in discussions about feeding tubes, for example:
  - survival
  - aspiration (breathing in of food)

In the next few pages, we will take about these outcomes so that you can have a better understanding of the advantages, disadvantages and other considerations about tube feeding.

栄養チューブをつけることによる合併症

本人が合併症を発症する確率についてある程度把握できるように、これまでに行われた研究結果を要約してみました。以下の数字は平均値（医学雑誌に発表された論文から抽出）で、人によって異なります。

合併症の種類	患者 100 人中、発症する可能性がある人数 *
感染症<br>• 軽度（皮膚）[3,5-10]<br>• 重度（生死にかかわる）[4,5,8,9]	100 人中 4 人<br>100 人中 1 人
出血<br>• 軽度（輸血なし）[3,4,7]<br>• 重度（輸血の必要性あり）[3,4,7]	100 人中 1 人未満<br>100 人中 0 人近く
一時的な下痢や腹痛[3,5,9,11]<br>一時的な嘔吐や吐き気[3,5,11]	100 人中 12 人<br>100 人中 9 人
チューブによる問題<br>• 軽度（抜け、詰まり、漏れ）[3-9]<br>• 重度（腸に穴があく）[3,4,6,8-10]	100 人中 4 人<br>100 人中 1 人未満
死亡<br>• チューブをつけたことが原因[5,8,9]	100 人中 1 人未満

＊ これらの値は胃ろうの場合だけです。空腸ろうにおける平均値は異なる可能性があります。

ご本人はどのくらい長生きできるのでしょうか？

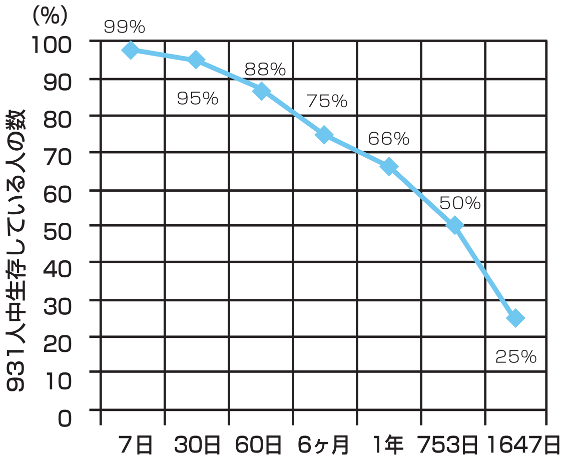

このグラフは栄養チューブをつけている 65 歳以上の高齢者 931 人当たり何人がチューブをつけてから 30 日後、60 日後、6 ヶ月後、一年後、二年後に生存しているかを示したものです。グラフに使われている数字は、日本における研究結果[31]を示したものです。特定の人の生存期間を確実に予測することは困難です。

栄養チューブをつけた人の経過を追った研究によると、以下の特徴がある人の生存期間は比較的短いことが明らかになっています。

- 超高齢者（85 歳以上）[7,10,20,23]
- 食物を誤嚥しやすい人[10]
- すでに栄養不良状態がすすんでいる人[7,15]
- がんと診断されている人[20,23,33]
- 血液検査の結果、炎症所見（CRP）と尿素窒素（BUN）の数値が高い人[31]

本人が延命を望む場合と望まない場合があり、これは、本人の生活の質、個人的な価値観や信条により異なる可能性があります。

Complications from feeding tube placement

We have tried to summarize the studies for you so that you can have some idea of the chances of your family member having a complication. The number below are average (taken from articles published in medical journals) which vary from patient to patient.

Type of complication	How many out of 100 patients might get it?
Infections	
minor (skin)	4 out of 100
major (life threatening)	1 out of 100
Bleeding	
minor (no transfusion)	less than 1 out of 100
major (need transfusion)	nearly 0 out of 100
Temporary diarrhea, cramping	12 out of 100
Temporary vomiting, nausea	9 out of 100
Tube problems	
minor (dislodgment, blockage, leaking)	4 out of 100
minor (perforation of bowel)	less than 1 out of 100
Death	
from putting the tube in	less than 1 out of 100

How long can I expect my family member to live?

This chart shows you how many out of 931 elderly patients who have feeding tubes will still be alive in 7days, 30days, 60days, 6months, 1year, 753days, and 1647days, after putting the tube in. The number on the chart are average over many studies. It is difficult to know for sure how long any one patient will live.

Few studies shows have shown that those with the following characteristics have a shorter survival:

- very old patients (over 85 years)
- patients who tend to aspirate (breathe in) their food
- patients who are already very undernourished
- patients with a previous diagnosis of malignancy
- patients with increased indicators of inflammation (i.e., C-reactive protein and blood urea nitrogen) are identified by a blood test

Prolonging life may or may not be what your family member would want. This may depend on his quality of life and personal values or beliefs.

チューブによる栄養法を用いた場合の「生活の質」について

海外では、チューブによる栄養法を用いた場合の本人の「生活の質」について、研究されていません。
日本では、胃ろうを作った認知症患者の家族を対象にし、胃ろうを作ったことは結果的によかったと思うかどうか、について調査 32 をしています。この調査の結果をみると、33 人中、60%の家族は「よかった」と答え、「よかったか、よくなかったか判断に迷う」と答えた方が 26.7%、「よくなかった」と答えた方が 13.3%いました。
死は、誰にでもおとずれるものであり、口から食べられなくなったら寿命を迎えつつある、という考え方もあります。命はただ延ばせばいいというものではなく、生きている間の生活の質をなるべく高く保つこと（本人の人生がより豊かで快適であること）が重要です。

栄養チューブをつけることで誤嚥（ごえん）は減るのでしょうか？

複数の研究結果[23,27,28] から栄養チューブをつけることで誤嚥を必ずしも防止できるとは限らないことが分かっています。これらの研究によると、栄養チューブをつける前に誤嚥したことがある患者の半数以上が、栄養チューブをつけた後でも誤嚥していました。平均すると、栄養チューブをつけた人 100 人中 16 人が誤嚥することになります[3,6,7,10]。

栄養チューブをつけるかどうかを判断する際に考慮すべき要素にはその他どのようなものがありますか？

嚥下障害がある脳卒中の患者では、脳卒中を発症してから早期に栄養チューブをつける方が、数週間待ってからつけるより回復しやすい可能性があります。[32]
周囲で起こっていることを全く認識しておらず、数ヶ月間、日常生活に必要なことを全て他人が面倒を見ている状態の方では、栄養チューブの有無に関わらず、状態の改善が認められる可能性は低くなります。[18]
栄養チューブをつけるか、つけていないかで、入所出来る施設が限定される可能性があります。医療チームとこの点について話し合う必要があります。
栄養チューブをつけている患者の中には栄養チューブを異物と感じ、イライラして落ち着かず、チューブを引き抜こうとする患者もいます。これを防止するため、入院している場合、医療チームが拘束や薬剤投与を提案することがあります。意思決定を行う代理人として、この判断に関わる必要があります。代理人の同意なしでこの判断がなされてはなりません。

Quality of life of patients who placing a feeding tube

There are no study regarding quality of life of patients who placing a feeding tube.

The perception of families of dementia patients who placing a feeding tube;

- 60% of 33 families answered 'good'
- 26.7% answered that they "wavered in judgment between 'good' and 'notgood'"
- 13.3% answered 'not good'

Death visits all of us and some believe that becoming incapable of oral ingestion is a sign that a person is approaching the end of their life. Life is not something that should simply be extended; it is important to maintain the highest possible quality of life while one is alive (in other words, that a person's life is more productive and comfortable).

What is aspiration and how dose it affect my family member?

It is clear from several case series that putting in a feeding tube will not necessarily stop a patient from aspirating. More than half of patients in these studies who aspirated before they were given a tube, still aspirated after they were given a tube. On average, 16 out of 100patients with a feeding tube will aspirate.

What other factors are important to consider when deciding about placing a feeding tube?

- Stroke patients who have swallowing problems may recover better if the feeding tube is placed earlier on in their illness, rather than waiting a few weeks.
- Patients who have been totally unaware of their surroundings and dependent on other to look after their basic needs for several months are less likely to improve, whether they have a feeding tube or not.
- Whether or not a patient gets a feeding tube may determine what kind of facility he can live in. You should discuss this with the health care team.
- Some patients with feeding tubes may become agitated and/or may try to pull the tube out. The health care team may suggest restraints or medications to stop the patient from doing this. As the substitute decision-maker, you should be involved in this decision. This should not happen without your consent.

チューブによる栄養法は中止できるのでしょうか？

栄養チューブをつけると決める前に、チューブの除去やチューブによる栄養法の中止に関する事柄を理解しておくとよいでしょう。

考慮すべき技術的な事項

栄養チューブが不要になった時、チューブを引き抜くことは技術的には簡単です。それには次の方法があり、医療者が行います。

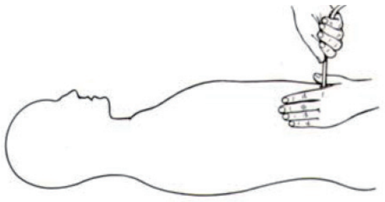

1）　引っ張ってチューブを抜きます。この方法でチューブは除去できるようになっています。安全で、痛みはほとんどありません。

もしくは

2）　体外でチューブを切断し、内視鏡を用いて口からチューブを取り除きます。

チューブによる栄養法の中止が考えられる理由

- 本人が普通に食事を取れるまでに回復した場合

もしくは

- 本人は回復せず、栄養チューブが本人にとって最善の処置ではなくなった場合
  この場合、チューブによる栄養法を中止するか否か医療チームとともに話し合いましょう。

栄養チューブの利点や欠点及び他に考慮すべき事項は何でしょうか？

利点

＋本人が再び食事が取れるまでに回復する可能性があります。
＋本人はより多くの栄養を取ることが出来ます。
＋口からは飲みにくい薬を栄養チューブから入れることができます。

欠点

－軽度もしくは重度の出血、感染、チューブによる問題、死亡などチューブによる栄養法による合併症が生じる可能性があります。
－本人が、栄養チューブを異物と感じて、イライラして落ち着かない可能性があります。
－栄養チューブにより介護を受ける場所が制限される可能性があります。高齢者を受け入れる施設（特別養護老人ホームなど）は医療者の数が少ないため、栄養チューブをつけた方を受け入れることが、難しい場合があります。一方、療養型病院などでは、栄養チューブをつけることを求められる場合があります。

他の考慮すべき事項

- 誤嚥しやすい人では、栄養チューブによって誤嚥は防止出来ません。
- 栄養チューブによって、本人の人生がより豊かになり快適に過ごせるようになる場合とそうでない場合があります。
- 意思決定を行うためのステップをふむことで、納得のいく意思決定を行いましょう。

Can tube feeding be discontinued?

Before you decide to put in the tube, you may want to think about what may be involved in deciding to remove the tube or stop tube-feeding at a later date.

Technical Considerations

It is technically easy to remove the tube by:

1) pulling it out using traction-the tube is designed to be removed this way-it is safe, and nearly painless or

2) cutting the tube on the outside, then using an endoscope to remove it through the mouth.

Possible reasons for discontinuing tube feeding

The patient may have improved enough to be able to eat normally or

The patient may not have improved and the tube may no longer be in their best interests

As a substitute decision-maker, it is your choice to stop tube feeding. You should discuss this decision with the patient's health care team.

What are the advantages, disadvantages and other considerations of feeding tube placement?

Advantage

patient may improve enough to be able to eat again

patient gets more nutrition

Disadvantage

complications from tube feeding, such as minor or major bleeding, infections, tube problems or death

may become agitated with the tube

feeding tube may limit where patient can receive care

Other Considerations

will not prevent aspiration in those who are likely to aspirate

certain factors are associated with decreased chances of survival

feeding tube may not improve quality of life

Steps to making the decision

治療の選択肢は？

介護対象となっている人に摂食・嚥下障害がある場合、医療チームは以下の選択肢を提案します。

サポーティブケア
＋
栄養チューブの設置

または　**サポーティブケアのみ**

サポーティブケアとは何でしょうか？

サポーティブケアとは、本人の生活の質を高めるケアともいえます。具体的には以下のものがあります。

１　可能な場合は、食事の介助をする
２　本人の不快感を取り除くための他の処置
３　輸液療法

1.　食事の介助

- 摂食障害のある高齢者では、食事の介助が出来る場合と出来ない場合があります。
- 栄養チューブをつけている人の中には、口からも食物を摂取できる人がいます。

食事の介助が出来るかどうかはどのように判断するのでしょうか？

- 医療チームのメンバー（例えば、医師、看護師、言語聴覚士など）が患者の食事の介助をしても安全かどうかを判断します。
- 特別な嚥下試験により、本人が無理なく摂取できる食物の硬度を調べる場合があります。

誰が本人の食事の介助をするのでしょうか？

- 訓練を受けた医療従事者（看護師、看護助手など）
- 家族、友人、ボランティアの人

どのように食事の介助をするのでしょうか？

- 摂食障害のある人の誤嚥を防止するために適切な食事介助方法が必要となります。

その方法には次のものがあります。

- 本人をベッドから起き上がらせます。
- 適切な軟らかさの食物を選びます。
- 本人が食物をうまく飲み込めず口の中に残している場合は、吸引をして取り除くことがあります。
- 食事の介助には１時間程度かけることがあります。

2.　摂食・嚥下障害にかかわらず、本人の不快感を取り除くための他の処置

- 本人の口をグリセリン綿棒や氷のかけらで湿らせます。
- 薬剤による痛みの抑制
- 呼吸障害がある場合には酸素吸入
- 便秘の治療
- 精神的もしくは情緒的なサポート
- 皮膚の手入れ

3.　輸液療法

- 点滴をすることもできます。摂取カロリーは、チューブを用いた栄養法に比べ低いです。主に水分を投与することができます。

What are my treatment choices?
Because the person in your care is having eating and/or swallowing problems, the health care team is offering the choice of:
supportive care plus placement of a feeding tube or
supportive care

What is supportive care?
Supportive care involves:
1. hand-feeding if possible
2. other treatments to keep the patient comfortable
3. fluid therapy

1. Hand-feeding
patients with eating problems who do not receive a feeding tube may or may not be able to be hand fed
some patients with a feeding tube may also be able to get some food by mouth
How is it decided if a patient can be hand-fed?
members of the health care team (for example, doctor, nurse, dietitian, speech and language pathologist, occupational therapist) will decide how safe it is to hand feed a patient
a special swallowing study may be done to see what consistency of food the patient can tolerate easily
Who hand feeds the patient?
trained health care professionals (nurse, nursing assistant or aide)
family, friends, volunteers
How are patients hand-fed?
proper feeding techniques are needed to help prevent patients with eating problems from aspirating. These techniques include from aspirating. These techniques include:
sitting her up in bed
choosing food of mouth when necessary
hand-feeding a meal can take as long as two hours

2. Other treatments to keep the patients comfortable
keeping the patient's mouth moist with a glycerin swab or ice chips
pain control, with medication
oxygen, for breathing problems
treatment of constipation
spiritual or emotional support
skin care

3. Fluid therapy
intravenous infusions can also be given
calorie intake is lower compared with tube feeding
water can mainly be administered

意思決定を行うためのステップ

① **本人はどのような状況に置かれていますか？**

- 摂食 (食べること) 障害の原因となっている病気が良くなる見込みはありますか？
- 栄養を供給するために栄養チューブは必要ですか？
- 栄養チューブに伴う特定の合併症（例えば軽度もしくは重度のチューブによる問題、出血、感染）についてどの程度ありそうですか？
- 本人が栄養チューブによってイライラして落ち着かない可能性があり、チューブをつけ続けるために拘束する必要がありそうですか？
- 栄養チューブをつけることにより、本人が生活できる場所が変わりますか？
- 本人は誤嚥していますか？
- 本人には生存の可能性が減少する特定の要因 (がんなど) がありますか？
- 栄養チューブは本人の生活の質にどんな影響を及ぼしそうですか？

② **本人は何を望むでしょう？**

- 栄養チューブなどの医療技術の利用について、本人はこれまでに意思（リビングウィルもしくは以前に語っていた事で）を表示したことがありますか？
- 終末期介護について本人はどのような信条や価値観を持っていますか？
- 本人が栄養チューブの利点と欠点を比較した場合、本人はどのような選択をすると思いますか？
- 本人にとって何が最善だと思いますか？

③ **本人に代わって決定するがあなたに与える影響**

- 罪悪感
- 他人からのプレッシャーを感じる
- 自分の個人的信条と患者の信条との間での葛藤
- 栄養チューブを継続することに関する将来的な判断についての不安

④ **決断する前に明確にしておかなければならない疑問点は？**

⑤ **誰が栄養チューブをつけることについての判断をすべきですか？**

⑥ **栄養チューブをつけることについて、総合的にどのような見解を持っていますか？**

意思決定を行う助けとなるノートを作成しました。書き込みながら、考えを整理していきましょう。

次の数ページに渡り、意思決定を代理で行った数例を紹介します。

これらの例は、ある判断方法が、正しいか間違っているかを示唆することを目的として挙げられている訳ではありません。

Steps to making the decision

1 What is your family member's situation?

- is the underlying condition causing the eating problem likely to get better?
- is the feeding tube needed to help provide nutrition?
- how concerned are you about specific complications of the feeding tube (such as minor or major tube problems. bleeding, infections)?
- is the patient likely to become agitated with the tube and need to be restrained to keep it in?
- will feeding tube placement make a difference as to where the patient can live?
- is the patient have any of the factors associated with decreased chances of survival?
- dose the patient have any of the factors associated with decreased chances of survival?
- how will the feeding tube affect quality of life?

2 What would your family member want?

- has she ever expressed her wishes (in a living will or previous discussion) about the use of medical technologies like feeding tubes?
- what are his beliefs and values about end-of-life care?
- if she could weigh the advantages and disadvantages, what do you think she would choose
- what do you feel is in your family member's best interests?

3 How the decision is affecting you:

- feeling of guilt
- feeling of pressure from others
- conflict between your personal beliefs and those of the patient
- worry about the future decisions regarding continuing with the tube

4 What questions need answering before you can decide?

5 Who should decide about planing the tube?

6 What is my overall "learning" about placing a feeding tube?

Over the next few pages, we will show you some examples of substitute decision makes like you.
The examples are not meant to suggest a right or wrong way to make the decision.

意思決定の方法例① 花子さんの場合

花子さんは数日前に突然、脳卒中を発症しました。

① 花子さんの健康状態

利　点	他の考慮すべき事項	欠　点
回復<br>不確実<br><br>栄養状態<br>- 栄養不良なし<br>- 食事介助は必要？<br>- 床ずれなし	生存の可能性<br>- 85 歳未満<br>- 栄養不良なし<br>- 悪性腫瘍なし<br><br>誤嚥<br>なし	合併症<br>リスクは小さい<br>興奮（イライラ）<br>可能性は低い<br>生活の場<br>チューブをつけると　長期介護施設へ移動することになります

生活の質
- 過去３ヶ月間の生活の質は良好
- チューブをつけることにより満足できる生活の質が得られますか？
- チューブの設置により花子さんが再び自立した生活を送ることが出来るなら彼女は望むでしょう

② 花子さんが望むと思われることは何ですか？

これまでの話し合い　あり

リビングウィル　なし

栄養チューブについての本人の考え

☐ ☑ ☐ ☐ ☐ ☐ ☐

賛成　　わからない　　反対

③ 判断があなたにどういう影響を与えますか？

罪悪感 - あまりない　　他人からのプレッシャー - あまりない
葛藤 - あまりない　　将来の不安 - 大きい

④ 決断する前に明確にして置かなければならない疑問点は何ですか？

花子さんが脳卒中から回復する見込みはありますか？これから数ヶ月間以内に回復しない場合、彼女がチューブの継続を望むとは思えません。その時点で、チューブを抜くことを決められますか？チューブの除去はどの程度難しいのですか？

⑤ 誰が栄養チューブをつけることについての判断をすべきですか？

花子さんがこれまでに行った意思表示を基にして、花子さんの医師と私が一緒に判断します。

⑥ 栄養チューブをつけることについて、総合的にどのような見解を持っていますか？

☐ ☑ ☐ ☐ ☐ ☐ ☐

チューブをつける　　わからない　　サポーティブケアのみ

Hanako's personal worksheet -Hanako had a sudden stroke a few days ago

1. Your family member's health situation

Advantages	Other considerations	Disadvantages
Improvement	Survival	Complication
unsure	under 85	small risk
	not malnourished	Agitation
	no malignancy	unlikely
Nutrition	Aspiration	Facility
not malnourished	no	tube will mean move to a
hand feeding?		chronic care facility
no bedsores		

Quality of Life
good quality of life in past 3months
will the tube provide an acceptable quality of life?
if tube will help her regain her independence

2.What would your family member want?

Previous discussion-yes
Living will- no

Patient's feeling about feeding tube
in favor unsure against

3. How is the decision affecting you?

guilt - not much
conflict - not much
pressure from pothers - not much
worry about future - a lot

4. What questions need answering before you can decide?
How likely is she to recover from the stroke? If she doesn't improve in the next couple of months I doubt Hanako would want to continue with the tube. Can we decide to remove it at that point? How hard is it to remove?

5. Who should decide about placing the tube?
Based on Hanako's previously expressed wishes, her doctor and I will decide together.

6. What is my overall "learning" about placing a feeding tube?

put in tube unsure supportive care only

意思決定の方法例② 太郎さんの場合

太郎さんは進行性アルツハイマー病を８年間患っています。

① 太郎さんの健康状態

利　点	他の考慮すべき事項	欠　点
回復<br>可能性は低い<br><br>栄養状態<br>- 非常に重い栄養失調<br>- 食事の介助は可能？<br>- 床ずれあり	生存の可能性<br>- 85 歳以上<br>- 非常に重い栄養失調<br>- 悪性腫瘍なし<br><br>誤嚥<br>あり	合併症<br>リスクは小さい<br>興奮（イライラ）<br>可能性あり<br>生活の場<br>チューブをつけると施設を移動することになるかは不明

生活の質
- 過去３ヶ月間の生活の質は不良
- チューブをつけることにより太郎さんにとって許容できる生活の質が得られる可能性は低い
- 主要な目的は不快感を除くこと

② 太郎さんが望むと思われることは何ですか？

これまでの話し合い　なし

リビングウィル　あり

栄養チューブについての本人の考え

賛成 ☐ ☐ ☐ ☐（わからない） ☐ ☐ ☑ 反対

③ 判断があなたにどういう影響を与えますか？

罪悪感 - 多少ある　　他人からのプレッシャー - あまりない
葛藤 - あまりない　　将来の不安 - あまりない

④ 決断する前に明確にして置かなければならない疑問点は何ですか？

チューブがないと太郎さんは空腹感やのどの渇きを感じますか？
太郎さんの介護施設では栄養チューブをつけた人を受け入れていますか？

⑤ 誰が栄養チューブをつけることについての判断をすべきですか？

太郎さんからこの状況でどうすればいいのかについての話はなかったので、太郎さんが望むと思われることを基に私が判断しなければなりません。太郎さんを長年にわたって診てきた医師と相談します。

⑥ 栄養チューブをつけることについて、総合的にどのような見解を持っていますか？

チューブをつける ☐ ☐ ☐ ☐（わからない） ☐ ☑ ☐ サポーティブケアのみ

Taro's personal worksheet -Taro has had progressive Alzheimer's disease for eight years.

1. Your family member's health situation

Advantages	Other considerations	Disadvantages
Improvement<br>unlikely	Survival<br>over 85<br>very malnourished<br>no malignancy	Complication<br>unlikely<br>Agitation<br>unlikely
Nutrition<br>very malnourished<br>hand feeding possible?<br>has bedsores	Aspiration<br>yes	Facility<br>unsure if tube will mean change in facility

Quality of life
poor quality of life in past 3months
unlikely that tube will provide acceptable quality of life to Taro
major goal is comfort

2.What would your family member want?

Previous discussion-no
Living will- yes

Patient's feeling about feeding tube

in favor	unsure	against

3. How is the decision affecting you?

guilt - somewhat
conflict - not much
pressure from pothers - not much
worry about future - not much

4. What questions need answering before you can decide?
Will Taro feel hunger and thirst without the tube?
Dose his nursing home accept tube-fed patients?

5. Who should decide about placing the tube?
Taro hasn't told me what to do in this situation, so I must decide for him based on what I think he would want. I'll talk it over with his doctor who has known him for a long time.

6. What is my overall "learning" about placing a feeding tube?

put in tube	unsure	supportive care only

意思決定の方法例③ 梅さんの場合

梅さんは 10 日前、重度の脳卒中を発症しました。
医師によると彼女は回復できないかもしれない、ということでした

① 梅さんの健康状態

利　点	他の考慮すべき事項	欠　点
回復<br>可能性は低い<br><br>栄養状態<br>- 栄養不良なし<br>- 食事介助は必要？<br>- 床ずれなし	生存の可能性<br>- 85 歳未満<br>- 栄養不良なし<br>- 悪性腫瘍なし<br><br>誤嚥<br>あり	合併症<br>リスクは小さい<br>興奮（イライラ）<br>不明<br>生活の場<br>チューブをつけると　別の施設に移動することになります

生活の質
- 過去３ヶ月間の生活の質は良好
- 梅さんが良好な生活の質を再び得る可能性は低いが、梅さんにとってそれが問題となるとは思えません
- 宗教的信条は梅さんにとって非常に重要なものです

② 梅さんが望むと思われることは何ですか？

これまでの話し合い　あり

リビングウィル　なし

栄養チューブについての本人の考え

☐ ☐ ☐ ☑ ☐ ☐ ☐

賛成　わからない　反対

③ 判断があなたにどういう影響を与えますか？

罪悪感 - 大きい　他人からのプレッシャー - 多少ある
葛藤 - 大きい。自分ならチューブを望みません。　将来の不安 - 多少ある

④ 決断する前に明確にして置かなければならない疑問点は何ですか？

どのような判断をすれば梅さんの宗教的信条が最も尊重されるでしょうか？

⑤ 誰が栄養チューブをつけることについての判断をすべきですか？

医師と梅さんをよく知る牧師と相談してから判断します。

⑥ 栄養チューブをつけることについて、総合的にどのような見解を持っていますか？

☐ ☐ ☐ ☑ ☐ ☐ ☐

チューブをつける　わからない　サポーティブケアのみ

Ume's personal worksheet -Ume had a big stroke 10 days ago.

1. Your family member's health situation

Advantages	Other considerations	Disadvantages
Improvement<br>unlikely	Survival<br>under 85<br>not malnourished<br>no malignancy	Complication<br>small risk<br>Agitation<br>unsure
Nutrition<br>not malnourished<br>hand feeding?<br>no bedsores	Aspiration<br>yes	Facility<br>tube will mean move<br>from her residence to a difference<br>facility

Quality of life
good quality of life in past 3months
it is unlikely she will return to that quality of life, but that may not matter to Ume
her religious beliefs are very important to her

2.What would your family member want?

Previous discussion- yes
Living will- no

Patient's feeling about feeding tube
in favor unsure against

3. How is the decision affecting you?

guilt - a lot
conflict - a lot, I wouldn't want a tube if I

pressure from others - somewhat
worry about future - somewhat

4. What questions need answering before you can decide?
What decision would best respect her religious beliefs?

5. Who should decide about placing the tube?
I will decide after talking to the doctor and Ume's pastor, who knows her well.

6. What is my overall "learning" about placing a feeding tube?

put in tube unsure supportive care only

資料編

チューブを用いた栄養法についての研究方法

結果について理解を深めるために、実施可能な研究方法には異なる種類があることを知っておく必要があります。基本的には 3 種類あります。

無作為化試験（むさくいかしけん）

- 栄養チューブをつけるかどうかはランダムに選んで決められます。
- 栄養チューブをつけた人を、栄養チューブをつけない人と比較できます。
- 確信性の高い結果が得られます。

（チューブを用いた栄養法の無作為化試験は実施されたことがありません。）

非無作為化試験（ひむさくいかしけん）

- 栄養チューブをつけることを選択した人と栄養チューブをつけない人を比較します。
- 栄養チューブをつけることを選択した人は、栄養チューブをつけない人と比較して、結果に影響を及ぼす様々な要因が異なっている可能性があります。
- 結果の確実性は、比較的低くなります。

症例検討

- 栄養チューブをつけた人のグループの経過観察を行います。

チューブを用いた栄養法についての研究結果（生存の可能性）

栄養チューブをつけた人と、つけていない同様の人のどちらの生存期間が長くなるかを比較した無作為化試験はありません。そのため、生存に関するこの質問に対する簡単な答えはありません。

高齢者養護施設において実施された非無作為化試験により、チューブによる栄養法の人とそうではない同様の人では、生存期間に差はないことが分かっています。しかし、チューブによる栄養法を行わなかったら、これらの人がどのくらい生存できていたのかは不明です。チューブによる栄養法を行った人は行わなかった人に比べて、病気が重かった可能性があります。

チューブを用いた栄養法についての研究結果（誤嚥の可能性）

栄養チューブをつけた人とつけていない人で誤嚥の発生率を比較した無作為化試験はありません。

栄養チューブをつけた人とつけていない人を比較した非無作為化試験では[14,26]、チューブをつけた人の方が、誤嚥しやすい傾向があることが認められています。しかし、これらの試験結果では、栄養チューブをつけたために誤嚥する可能性が高くなったのか、誤嚥しやすい人が栄養チューブをつけることが多いのかは明らかでありません。

Ranking studies about tube feeding

In order to learn about health outcomes, you need to understand about the different types of research studies that can understand about the different types of research studies that can be done. There are basically three kinds:

Randomized Trials

whether or not someone gets a feeding tube is based on a toss of a coin

patients with a feeding tube are comparable to patients without a feeding tube

more confident in the results (There are no randomized trials of tube feeding)

Non-Randomized Trials

patients who have chosen to have feeding tubes are compared to patients without feeding tubes

tube fed patients may be different from patients without feeding tubes in ways that may affect the outcomes

less confident in the results

Case Series

A group of patients with feeding tubes are followed over time to see how they do

Will putting in a feeding tube increase the patient's chance of survival?

There are no randomized trials comparing similar patients with and without feeding tubes to see who lived longer. Because of this, there is no straightforward answer to this question of survival.

Non-randomized trials in nursing homes have found that tube fed patients do not live longer than similar patients without feeding tubes. However, it is not clear how long these patients would have lived if they had never been given a feeding tube. It could be that patients who are given tubes are sicker than patients who are not given tubes.

What is aspiration and how dose it affect my family member?

There are no randomized trials comparing the chances of aspiration in patients with and without feeding tubes. Non randomized trials comparing patients with and without feeding tubes show that patients with tubes are more likely to be aspirators. However, it is not clear from the studies if getting a feeding tube increases the chances of aspirating, or whether being an aspirator increase the chances of getting a feeding tube.

メモと疑問点

Notes and questions

参考文献

References

1. Buchanan A. Deciding for others. Milbank Quarterly 1986; 64(suppl 2):17-94.
2. McCann RM, Hill WJ, Groth-Junker A. Comfort care for terminally ill patients: the appropriate use of nutrition and hydration. JAMA 1994;272:1263-6.
3. Bourdel-Marchasson I, Dumas F, Pinganaud G, Emeriau J-P, Decamps A. Audit of percutaneous endoscopic gastrostomy in long-term enteral feeding in a nursing home. International Journal for Quality in Health Care 1997; 9(4):297-302.
4. Grant JP. Percutaneous endoscopic gastrostomy. Initial placement by single endoscopic technique and long-term follow-up. Annals of Surgery 1993; 217(2):168-174.
5. Hull MA, Rawlings J, Murray FE, Filed J, McIntyre AS, Mahida YR Hawkey CJ, Allison SP. Audit of outcome of long-term enteral nutrition by percutaneous endoscopic gastrostomy. The Lancet 1993; 341(April 3):869-872.
6. James A, Kapur K, Hawthorne AB. Long-term outcome of percutaneous endoscopic gastrostomy feeding in patients with dysphagic stroke. Age and Ageing 1998; 27:671-676.
7. Kaw M, Sekas G. Long-term follow-up of consequences of percutaneous endoscopic gastrostomy (PEG) tubes in nursing home patients. Digestive Diseases and Sciences 1994; 39(4):738-743.
8. Raha S, Woodhouse K. The use of percutaneous endoscopic gastrostomy (PEG) in 161 consecutive elderly patients. Age and Ageing 1994; 23:162-163.
9. Sali A, Wong P-T, Read A, McQillan T, Conboy D. Percutaneous endoscopic gastrostomy: The heidelberg repatriation hospital experience. Australian and New Zealand Journal of Surgery 1993; 63:545-550.
10. Light VL, Slezak FA, Porter JA, Gerson LW, McCord G. Predictive factors for early mortality after percutaneous endoscopic gastrostomy. Gastrointestinal Endoscopy 1995; 42(4);330-335.
11. Panos MZ, Moran A, Reilly T, Wallis PJW, Wears R, Chesner IM. Percutaneous endoscopic gastrostomy in a general hospital: prospective evaluation of indications, outcome, and randomised comparison of two tube designs. Gut 1994; 35:1551-1556.
12. Cowen ME, Simpson SL, Vettese TE. Survival estimates for patients with abnormal swallowing studies. Journal of General Internal Medicine 1997;12(February);88-94.
13. Mitchell SL, Kiely DK, Lipsitz LA. Does artificial enteral nutrition prolong the survival of institutionalized elders with chewing and swallowing problems? Journal of Gerontology 1998; 53A(no.3):M207- M213.
14. Mitchell SL, Kiely DK, Lipsitz LA. The risk factors and impact on survival of feeding tube placement in nursing home residents with severe cognitive impairment. Archives of Internal Medicine 1997; 157:327-332.
15. Friedenberg F, Jensen G, Gujral N, Braitman LE, Levine GM. Serum albumin is predictive of 30-day survival after percutaneous endoscopic gastrostomy. Journal of Parenteral and Enteral Nutrition 1997; 21(2):72-74.
16. Horton WL, Colwell DL, Burlon DT. Experience with percutaneous endoscopic gastrostomy in a community hospital. American Journal of Gastroenterology 1991; 86:168-169.
17. Jarnagin WR, Duh QY, Mulvihill SJ, Ridge JA, Schrock TR, Way LW. The efficacy and limitations of percutaneous endoscopic gastrostomy. Archives of Surgery 1992; 127:261-264.
18. Fay DE, Popausky M, Gruber M, Lance P. Long-term enteral feeding: a retrospective comparison of delivery via percutaneous endoscopic gastrostomy and nasoenteric tubes. American Journal of Gastroenterology 1991; 86:1604-1609.
19. Kadakia LTCSC, Sullivan HO, Starnes E. Percutaneous endoscopic gastrostomy or jejunostomy and the incidence of aspiration in 79 patients. The American Journal of Surgery 1992; 164(August):114-118.
20. Rabenek L, Wray NP, Petersen NJ. Long-term outcomes of patients receiving percutaneous endoscopic gastrostomy tubes. Journal of General Internal Medicine 1996; 11:287-293.
21. Samii Am, Suguitan EA. Comparison of operative gastostomy with percutaneous endoscopic gastrostomy. Mil Med 1990; 155:534-535.
22. Stuart SP, Tiley EH, Boland JP. Feeding gastrostomy: a critical review of its indications and mortality rate. Southern Medical Journal 1993; 86:1689-172.
23. Taylor CA, Larson DE, Ballard DJ, Bergstrom LR, Silverstein MD, Zinsmeister AR, DiMagno EP. Predictors of outcome after percutaneous endoscopic gastrostomy: A community-based study. Mayo Clinic Proceedings 1992; 67:1042-1049.
24. Tealey AR. Percutaneous endoscopic gastrostomy in the elderly. Gastroenterology Nursing 1994; February:151-157.
25. Wolfsen HC, Kozarek RA, Ball TJ. Long-term survival in patients undergoing percutaneous endoscopic gastrostomy and jejunostomy. American Journal of Gastroenterology 1990; 85:1120-1122.
26. Pick N, McDonald A, Bennett N, Litche M, Dietsche L, Legerwood R, Spurgas R, LaForce FM. Pulmonary aspiration in a long-term care setting: clinical and laboratory observations and an analysis of risk factors. Journal of the American Geriatrics Society 1996; 44:764-798
27. Patel PH, Thomas E. Risk factors for pneumonia after percutaneous endoscopic gastrostomy. Journal of Clinical Gastroenterology 1990; 12 (4):389-392.
28. Finucane TE, Bynum JD. Use of tube feeding to prevent aspiration pneumonia. The Lancet 1996;348(9039):1421-1441.
29. Wanklyn P, Cox N, Belfield P. Outcome in patients who require a gastrostomy after stroke. Age and Ageing 1995; 24:510-514.
30. Fisman DN, Levy AR, Gifford DR, Tamblyn R. Survival after percutaneous endoscopic gastrostomy among older residents of Quebec. Journal of the American Geriatrics Society 1999; 47:349-353.
31. Yutaka Suzuki et al. Survival of geriatric patients after percutaneous endscopic gastrostomy in Japan. World Journal of Gastroenterology 2010:16(40): 5084-5091
32. 日本老年医学会事業（平成22年度厚生労働省老健局老人保健健康増進等事業）：認知用末期患者に対する人工的な栄養・水分補給法の導入・差し控え・中止に関するガイドライン作成へ向けた検討.日本老年医学会

32. Japan Geriatrics Society enterprise, Japan Geriatrics Society 2010: **Examination of guideline creation regarding the introduction, continuation, and cessation of artificial nutrition and hydration to a terminally ill patient with dementia** [http://www.jpn-geriat-soc.or.jp/info/topics/pdf/jgs_ahn_gl_2012.pdf]

"Making choice:Long term feeding tube placement in elderly patients".
Developers:
SL Mitchell MD MPH FRCPC (Geriatrics Medicine, Epidemiology)
JM Tetroe MA (Health Research)
AM O'connor RN PhD (Nursing, Epidemiology)
A Rostom MD FRCPC (Gastroenterology, Epidemiology)
C Villeneuve BSc RD (Dietitian)
B Hall RN BScN (Geriatric Nursing)

"Making choice:Long term feeding tube placement in elderly patients" Japanese version
Developer: Yumiko Kuraoka
This booklet was supported by MEXT KAKENHI (Grant Number 22890189,25862123).

First eddition：May 2011
Second eddition：May 2013

この小冊子は、Ottawa Health Research Institute の
SL Mitchell, MD MPH FRCPC（老年医学、疫学）、
JM Tetroe, MA（健康研究）、
AM O’　Connor, RN PhD（看護、疫学）、
A Rostom, MD FRCPC（消化器病学、疫学）、
A Villeneuve, BSc RD（栄養士）、
B Hall, RN BscN　（老人看護）
によって開発された「Making Choice：Long Term Feeding　Tube Placement in Elderly Patients」を参考に聖路加看護大学　倉岡有美子が作成した。
本小冊子は、以下の２つの日本学術振興会（文部科学省）の研究助成金によって制作した。

- 研究活動スタート支援「経口摂取に替わる栄養管理の導入を検討する患者・家族の意思決定支援ガイドの開発」（研究代表者　倉岡有美子、平成 22～ 24 年）課題番号 22890189
- 若手研究（B）「胃ろう造設を検討する患者の家族の意思決定支援ガイドの普及と評価」（研究代表者　倉岡有美子、平成 25～27 年）課題番号 25862123

2011 年５月　第１版
2013 年５月　第２版
